# Psychotropic drug initiation or increased dosage and the acute risk of falls: a prospective cohort study of nursing home residents

**DOI:** 10.1186/1471-2318-13-19

**Published:** 2013-02-22

**Authors:** Murray A Echt, Elizabeth J Samelson, Marian T Hannan, Alyssa B Dufour, Sarah D Berry

**Affiliations:** 1 Institute for Aging Research, Hebrew SeniorLife, 1200 Centre Street, Boston, MA, 02131, USA; 2 SUNY Downstate College of Medicine, 450 Clarkson Avenue, Brooklyn, NY, 11203, USA; 3 Harvard Medical School, Boston, MA, USA

**Keywords:** Falls, Nursing home, Psychotropic drug change

## Abstract

**Background:**

Previous studies suggest that psychotropic drug changes may signal an acute period of time whereby a person is highly vulnerable to fall. It is unknown whether certain classes of psychotropic agents are less safe with respect to the acute risk of falls. Our purpose was to compare fall rates in the 7 days following a change of an antidepressant, antipsychotic, or benzodiazepine. We also identified specific times when residents are at high risk for falls with respect to a psychotropic drug change.

**Methods:**

Residents in our one-year study included 851 long term care residents from two nursing home facilities in Boston, MA, U.S.A. (May 2010 - May 2011). Drug changes (i.e., new prescriptions or increased dose of a previously used drug) were ascertained using the computerized provider order entry system, whereas falls were ascertained by incident reports. Negative binomial regression was used to compare the rate of falls following a drug change between medication classes. Further, we calculated the rate of falls for each of the 7 days before and 7 days after a psychotropic drug change.

**Results:**

Forty-eight percent of residents were prescribed a new prescription or increased dose of a psychotropic drug during the study. The rate of falls was similar in the 7 days following a change to a SSRI versus non-SSRI antidepressant (11.9 versus 14.4 falls/1,000 person years; p = 0.58), a typical versus an atypical antipsychotic (25.4 versus 17.1 falls/1,000 person years; p = 0.10), or a short versus long acting benzodiazepine (15.2 versus 13.9 falls/1,000 person years; p = 0.23). Fall risk was highest on day 4 before the drug change (19.0 falls/1,000 person days), on the day of the drug change through 2 days after the drug change (17.6-20.3 falls/1,000 person days), and 5-6 days after the drug change (17.6-19.0 falls/1,000 person days).

**Conclusions:**

In the nursing home, risk of falls was similar following a psychotropic drug change of any class. We observed higher fall risk in the days before, but mostly after the drug change. We recommend that nursing home residents be closely monitored following a psychotropic drug change in an effort to reduce falls.

## Background

Falls in the nursing home are a serious health concern. As many as 3 out of 4 nursing home residents fall each year [1]. Nearly 10% of these falls result in fracture or serious injury [2]. The average cost per fall in the nursing home is $1,892 [3]. Even falls that do not result in injury can diminish self-confidence and cause loss of function [4]. Efforts to decrease falls in nursing home residents are needed to protect lives and reduce costs.

Psychotropic medications are associated with a 30-70% increased risk of falls [5,6]. Despite awareness that these medications may predispose an elderly person to fall, psychotropic drugs remain widely used (often for life) in the nursing home setting. It is important then, to identify specific time points or triggering events for falls among psychotropic drug users in order to target fall prevention efforts effectively.

Previous studies suggest that a medication change may signal an acute period of time whereby a nursing home resident is highly vulnerable to fall. For example, two small studies of nursing home residents found a 3–5 fold increased risk of falls within 3 days of a new prescription for a drug that affects the central nervous system [7,8]. It is unknown whether certain classes of psychotropic agents are less safe with respect to the acute risk of falls. Our objective was to compare the rate of falls in the 7 days following an antidepressant, antipsychotic, or benzodiazepine prescription drug change in a large, prospective cohort of long term care residents. We further described the rate of falls in the 7 days before and the 7 days after a drug change, in order to identify specific times when psychotropic medication users are particularly vulnerable to fall.

## Methods

### Participants

Residents in our year-long study included all long-stay residents of two nursing home facilities (Boston, MA, U.S.A.) between May 29, 2010 - May 28, 2011. Residents admitted to the short term rehabilitation units were not included. This population represents a dynamic cohort, as new residents are admitted and others are rehabilitated or pass away. This study was approved by the Hebrew SeniorLife IRB (Protocol #08-015). Informed consent was not required.

### Medication changes

Information on psychotropic drug changes was obtained using a computerized provider order entry system, MEDITECH™. A change was defined as either a new prescription or an increased dose of a currently prescribed psychotropic medication. A *new prescription* was defined as any prescription that was not used in the past 90 days on an as needed or scheduled basis. Changing drugs within a class of medications (i.e. replacing paroxetine with sertraline) was categorized as a new prescription. A *dose increase* was defined as an increase in the cumulative daily dose of a currently used medication or a medication that was prescribed in the past 90 days. A prescription ordered on the day of admission, started and stopped on the same day, and prescription renewals were not included as a drug change.

We used the American Hospital Formulary Services (AHFS Pharmacologic-Therapeutic Classification System) to classify medications as antidepressants (tricyclic, selective serotonin reuptake inhibitor (SSRI), miscelaneous), antipsychotics (typical and atypical), or benzodiazepines (short and long acting) [9]. We combined tricyclic and miscellaneous antidepressants in the category ‘non-SSRI antidepressants’ because there were too few tricyclic antidepressant changes (n = 9) to consider separately. Examples of non-SSRI antidepressants include nortriptyline, trazodone, mirtazapine, bupropion, and venlafaxine.

### Other variables

Information on cognitive status, pain, depression, and clinical characteristics that could be associated with the receipt of a psychotropic medication was ascertained using the Minimum Data Set (MDS) assessment performed closest to study entry (mean 35 days after study entry; range 0 days- 248 days after study entry). The MDS is a federally mandated assessment used to standardize detection of quality indicators in long term care facilities [10]. Cognitive impairment was classified as none or mild (0–1), moderate (2–4), or severe (5–6) using a validated Cognitive Performance Scale [11]. Pain was assessed using the Visual Analogue Scale and categorized as none (0), mild (1), or moderate to severe (2–3) [12]. Depression was assessed by a validated MDS Depression Rating Scale [13], and categorized as none (score 0), mild to moderate (score 1–2), or severe (score 3+). Clinical characteristics that could be associated with the receipt of a psychotropic medication including sadness, wandering, verbal or physical abuse, socially inappropriate behavior, and resistance to care were described as present or absent in the 7 days prior to completion of the MDS.

### Falls

Falls were ascertained prospectively through the facilities’ computerized incident reports. Nursing staff are trained annually on proper incident reporting. A fall was defined as accidentally coming to rest on the ground or lower surface, such as a chair.

### Statistical analysis

We compared characteristics between residents that never used psychotropic drugs, residents with chronic use of psychotropic drugs without a medication change, and residents with a psychotropic drug change during follow-up using ANOVA for continuous variables, and the Chi-squared test for categorical variables.

To calculate the rate of falls following a psychotropic drug change, we divided the total number of falls that occurred in the 7 days after a psychotropic drug change by the person days following the drug change. We used negative binomial regression adjusting for individual effects to compare the rates of falls following a drug change between classes of psychotropic medications [14]. Fully adjusted models considered age, sex, cognitive impairment, functional status, and pain. We planned to include history of falls in our models, but this resulted in unstable estimates. We believe this is because the majority of residents with a recurrent fall during the 7 days following a drug change were the same residents with a history of falls. Our models already included a random patient effect, and including both the random patient effect and the highly correlated exposure of history of falls was not possible.

We described the rate of falls for each of the 7 days before and 7 days after a psychotropic drug change (no formal statistical test). We excluded drug changes that occurred ≤ 14 days apart in the same participant (n = 283), in order to standardize time with respect to the date of the drug change. We do not present daily fall rates by medication class separately because the number of falls on an individual day was small when drugs were separated, and similar trends were observed for each class of medications.

## Results

During the one year study, 851 nursing home residents contributed a mean of 262 person-days of follow-up. Mean age of residents was 87 years and 75% were female. Forty-eight percent were prescribed a new psychotropic medication or an increased dose of a psychotropic medication. There was little difference in age, sex, and cognitive status of residents with a psychotropic drug change as compared to residents who used psychotropic medications without change or residents who never used psychotropic medications (Table 1). Residents with a psychotropic drug change were more likely to have moderate to severe pain as compared with residents who used psychotropic drugs without change (Table 1; 18.0% versus 11.9%, p < 0.001). The proportion of residents with ≥ 2 falls during follow-up was greater among residents with a psychotropic drug change as compared to psychotropic users without change (41.0% versus 22.5%, p < 0.001).

**Table 1:** Characteristics of 851 long term care residents as categorized by whether a psychotropic drug change occurred during follow-up (%, unless indicated)

	**Psychotropic drug change**	**Psychotropic user without a drug**	**No psychotropic medication use**	**P-value**
	**(n = 405)**	**change (n = 289)**	**(n = 157)**	
**Mean age (years, ± S.D.)**	87.4 ± 8.8	86.9 ± 8.5	87.1 ± 10.2	0.76
**Female**	75.1	73.0	75.8	0.76
**Cognition**				
**Normal or mild impairment**	20.1	20.6	29.0	
**Moderate impairment**	58.6	52.4	44.7	
**Severe impairment**	21.3	27.1	26.3	0.03
**Pain**				
**None**	55.6	66.4	74.5	
**Mild**	26.4	22.0	13.1	
**Moderate to severe**	18.0	11.6	12.4	<0.001
**Depression**				
**None**	41.4	60.5	74.2	
**Mild to moderate**	26.3	20.7	15.9	
**Severe**	32.3	18.8	9.9	<0.001
**Fallers during 1 year follow-up**				
**1 fall**	23.5	18.7	13.4	
**≥ 2 falls**	41.0	22.5	12.1	<0.001
**Rate of falls during 1 year follow-up (per 1,000 person-days)**	7.6	4.1	2.0	<0.001
**Mean follow-up (days, ± S.D.)**	268 ± 121	258 ± 136	255 ± 132	0.47

During follow-up there were 407 new prescriptions for a psychotropic medication and 616 dose increases of a psychotropic medication (Table 2). The total number of new prescriptions was highest for short acting benzodiazepines (n = 160) and lowest for typical antipsychotics and long acting benzodiazepines (n = 18 each). Dose increases occurred most frequently with atypical antipsychotics (n = 194) and least frequently among typical antipsychotics (n = 27) and long acting benzodiazepines (n = 23). The most common clinical characteristics reported among residents that received an antidepressant change were sadness (60%) and resistance to care (25%). Among residents with an antipsychotic change, sadness (70%), socially inappropriate behaviors (32%), resistance to care (36%), and verbal abuse (28%) were commonly reported, whereas residents with a benzodiazepine change were often characterized as sad (59%) or resistive to care (28%).

**Table 2:** Number of psychotropic drug changes and rate of falls in the 7 days following the psychotropic drug change among 851 long term care residents over one year of follow-up

**Psychotropic medication**	**New prescription**			**Dose increase**			**Combined medication change**		
	**# of new prescriptions**	**# falls in the 7 days after the med change**	Rate of falls in the 7 days after the med change*	**# of dose increases**	**# Falls in the 7 days after the med change**	Rate of falls in the 7 days after the med change*	**# of drug changes**	**# Falls in the 7 days after the drug change**	Rate of falls in the 7 days after the drug change*
**Antidepressants**	155	19	17.5	221	17	11.0	376	36	13.7
**SSRIs**	46	4	12.4	62	5	11.5	108	9	11.9
**Non-SSRIs**	109	15	19.7	159	12	10.8	268	27	14.4
**Antipsychotics**	74	7	13.5	221	31	20.0	295	38	18.4
**Typical**	18	2	15.9	27	6	31.7	45	8	25.4
**Atypical**	56	5	12.8	194	25	18.4	250	30	17.1
**Benzodiazepines**	178	18	14.4	174	19	15.6	352	37	15.0
**Long-Acting**	18	2	15.9	23	2	12.4	41	4	13.9
**Short-Acting**	160	16	14.3	151	17	16.1	311	33	15.2

* per 1,000 person days.

Among 851 residents, 420 residents experienced a total of 1,205 falls during the study period (5.4 falls/1,000 person days). The rate of falls was similar in the 7 days following a psychotropic drug change of any kind (Table 2; p ≥ 0.1 for all comparisons). Specifically, there was no difference in the rate of falls following a change to a SSRI versus non-SSRI antidepressant (11.9 versus 14.4 falls/1,000 person years; p = 0.58), a typical versus an atypical antipsychotic (25.4 versus 17.1 falls/1,000 person years; p = 0.10), or a short versus long acting benzodiazepine (15.2 versus 13.9 falls/1,000 person years; p = 0.23).

In the 7 days before and 7 days after a psychotropic drug change, 70 residents fell once and 24 residents fell multiple times (range 2–4 falls). Of the recurrent fallers, 13 fell in both the 7 days before and 7 days after the drug change. Three residents fell multiple times in the week before the drug change, whereas 7 residents fell multiple times in the week after the drug change. One resident fell twice on the day of the drug change.

**Figure 1:**
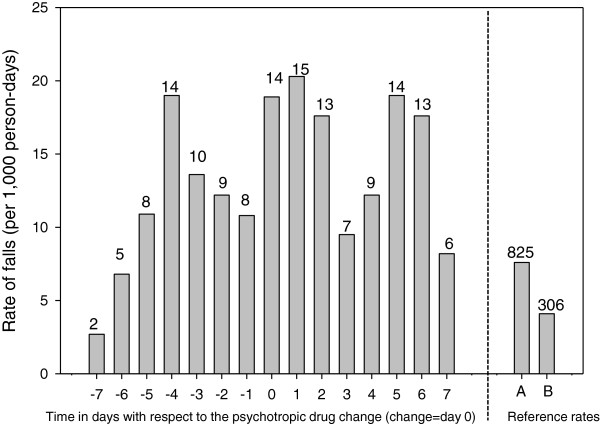
**Distribution of the rate of falls in nursing home residents with respect to a psychotropic drug change.** Negative values indicate days prior to the drug change, whereas positive values indicate days after the drug change. The number of falls is presented above each bar graph. Fall risk was greatest: 1) 4 days prior to the drug change, 2) on the day of the drug change through 2 days after the drug change, 3) 5–6 days after the drug change. For comparison, we present **A**) the annual rate of falls among psychotropic users with a drug change during one-year follow-up, **B**) the annual rate of falls among psychotropic users without a drug change during one-year follow-up.

The rate of falls was elevated in the days before and after a psychotropic drug change as compared with the rate of falls among psychotropic drug users that did not experience a medication change during follow-up, or compared with the annual rate of falls among psychotropic drug users that experienced a drug change (Figure 1). Specifically, risk of falls was elevated on day 4 before the drug change (19.0 falls/1,000 person days). Risk was also elevated on the day of the drug change through 2 days after the drug change (17.6-20.3 falls/1,000 person days), and days 5 to 6 after the drug change (19.0 and 17.6 falls/1,000 person days). On day 7 before the drug change and day 7 after the drug change, rates of falls were somewhat lower (2.7 and 8.2/1000 person days, respectively). In comparison, the annual rate of falls among psychotropic users with a drug change over the one year study was 7.6 /1,000 person days, and the annual rate of falls among psychotropic users without a drug change was 4.1/1,000 person days.

## Discussion

Our study suggests that there may be no acute protection afforded with respect to falls risk by selecting a newer versus older class of antidepressants or antipsychotics or a short acting versus long acting benzodiazepine. Previous studies have determined that the long term risk of falls and injurious falls was no different among users of tricyclic versus SSRI antidepressants [15], or among users of typical versus atypical antipsychotics [16]. Similarly, use of both short and long acting benzodiazepines has been associated with an equally elevated longitudinal risk of falls in community dwellers [17,18]. Thus, there is no evidence to support selection of psychotropic agents based on risk of falls.

Our finding that there was a high risk of falls in the days immediately following a psychotropic drug change is consistent with other studies. Using a self-controlled, case-crossover approach, Sorock and Neutel both found that the fall risk in nursing home residents was maximally increased within 2–3 days of a change in a medication that affects the central nervous system [7,8]. Previous work by our group showed that changes in specifically non-SSRI antidepressant prescriptions increased the risk of falls within 2 days, with a subsequent decreased risk of falls over the next 5 days [19]. Zint et al. concluded that in community dwellers, hip fracture risk associated with benzodiazepines was highest in users within 14 days of initiation [20].

Nonetheless, it is possible that the high rates of falls we observed are due to random variation in falls rates or because residents with psychotropic drug changes have more risk factors for falls, such as orthostatic hypotension, muscle weakness, or prior falls, predisposing them to fall. We did not have information on orthostatic hypotension or muscle weakness in order to adjust for these characteristics in our models. Although we had information on history of falls, this characteristic was not used in the final models due to statistical instability when we included both history of falls and an individual effect in the same model. When we included in our model a history of falls rather than a random patient effect, the associations between psychotropic drug changes and rate of falls did not change. We do not believe that any of these characteristics would fully account for the markedly elevated risk of falls we observed following a drug change. The rate of falls in the 7 days following an antidepressant, antipsychotic, or benzodiazepine drug change ranged from 13.7 to 18.4/1,000 person days. This is much greater than the annual rate of falls among residents that used psychotropic medications without change (4.1/1,000 person days) or the annual rate of falls among residents with a psychotropic drug change (7.1/1,000 person days).

While we observed that the rate of falls was highest one day following a psychotropic drug change, rates of falls were also elevated 3 to 4 days preceding the drug change. This suggests that the underlying medical condition for which the medication was prescribed also contributed to the increased risk of falls. In the nursing home, psychotropic medication changes are often made in an effort to manage psychiatric symptoms in persons with dementia, such as wandering, combativeness, and insomnia. Behavioral symptoms may begin days before pharmacologic treatment is selected resulting in an increased risk of falls before drug initiation.

Separating the effects of a drug from the effects of the underlying medical condition can be challenging. In our study, exposure to a psychotropic drug change was highly correlated with dementia with psychiatric symptoms. Attempts to adjust for psychiatric symptoms using a traditional model or propensity scores would have been of limited utility given the very high correlation between drug exposure and the psychiatric conditions [21]. Instead, we used a comparator medication strategy to determine if falls risk differed between classes of medications that are used to treat similar medical conditions. An alternative approach would have been a stratified or restrictive analysis, grouping residents with similar acute symptoms together [21]. One could test the hypothesis that it is the medical illness rather than the drug causing the observed risk by repeating the analysis with an alternative outcome that is known to have no association with the drug of interest [22]. We did not have an adequate sample size or precise information on other acute illnesses in order to use these strategies. Our study suggests that the rate of falls may be elevated in the days surrounding a psychotropic drug change. While it may be impractical to distinguish between the effects of the drug and the underlying medical condition, the implications of our findings remain: attention should be given to residents experiencing a psychotropic drug change in an effort to prevent falls.

Strengths of this study include a large, prospective cohort of long term care residents with complete information on medications and falls. Additionally our study addresses an important clinical question of whether certain psychotropic agents are less safe with respect to the acute risk of falls.

This study also has several potential limitations. First, we had small numbers of falls on individual days with respect to the drug change. While this may result in some variability of fall rates between days, overall trends were observed. Second, we were unable to consider the sizes of all new prescriptions and increased doses separately, and we did not consider concomitant medication changes given the limited number of falls in our study. For example, in our one-year study, 376 antidepressant changes occurred with only 36 falls within 7 days of the drug changes. Only a small fraction of antidepressant changes were associated with a concomitant benzodiazepine or antipsychotic drug change. Hanlon et al. found that risk of falling increases with the number and dosage of psychotropic medications [23], and it is possible that acute fall rates were underestimated in our study among residents who received a higher dose or multiple psychotropic medications. We recommend that future studies with a longer period of follow-up consider the effects of dose and concomitant drug changes on the risk of falls.

Third, information was not available on decreases or discontinuations in dosage so that we were not able to examine the effect of psychotropic drug reductions on the acute risk of falls. Withdrawal syndromes are possible with discontinuation of psychotropic medications, and could potentially result in an acute increased risk of falls. It is unlikely that decreases are a common cause of falls as previous studies suggest withdrawal of psychotropic medications reduces the risk of falls [24,25]. Nonetheless, long term benefits of withdrawing psychotropic agents are not mutually exclusive with the possibility of an acute risk of falling in the days after a decrease or discontinuation. Fourth, we did not have resident specific information on days spent outside the facility. We expect this number would be low, and accounting for days outside the facility would likely increase the rates of falls only slightly.

Finally, our study was conducted in two facilities operated by a single organization. In comparison with other facilities in the U.S., the proportion of residents prescribed an antidepressant or benzodiazepine was greater than the national average, while the proportion of residents prescribed an antipsychotic was similar to the national average [26,27]. In other countries, the prevalence of psychotropic drug use may be lower. The absolute effect of psychotropic drug changes on falls may be more or less significant depending on the frequency of use.

## Conclusions

We found the risk of falls in the 7 days following a psychotropic drug change is similar for antidepressants, antipsychotics, or benzodiazepines. Changes in psychotropic drugs are associated with an elevated risk of falls 4 days before the drug change, likely due to underlying psychiatric symptoms. Nursing home residents are also at an increased risk of falling in the days immediately following a psychotropic drug change, likely due to the effects of the drug itself and the medical illness. Increased staff awareness might help to reduce falls during this time. We suggest that nursing home residents should be monitored closely in the days following a psychotropic drug change in an effort to reduce falls.

## Competing interest

The authors declare they have no conflicts of interest.

## Authors’ contributions

MAE helped with the acquisition of the data, participated in analysis and interpretation of the data, and drafted the manuscript. EJS and MTH participated in the analysis and interpretation of the results, and provided critical revisions to the current manuscript. SDB conceived of the study idea, was responsible for acquiring the data, conducted all programming analyses, participated in the interpretation of the results, and provided critical revisions to the current manuscript. AD participated in the interpretation of results and provided critical revisions to the current manuscript. All authors read and approved the final manuscript.

## Pre-publication history

The pre-publication history for this paper can be accessed here:

http://www.biomedcentral.com/1471-2318/13/19/prepub
